# The Pitfall of Ganglioneuroblastoma-Nodular Diagnosis: Clinical and Imaging Considerations over a Rare Bifocal Sporadic Case

**DOI:** 10.3390/diagnostics12123221

**Published:** 2022-12-19

**Authors:** Claudio Montante, Francesco Fabozzi, Maria Felicia Villani, Maria Luisa D’Andrea, Alessandra Stracuzzi, Gian Luigi Natali, Giada Del Baldo, Francesca Del Bufalo, Maria Carmen Garganese, Annalisa Serra, Paolo Tomà, Rita Alaggio, Sabina Vennarini, Giovanna Stefania Colafati, Angela Mastronuzzi, Maria Antonietta De Ioris

**Affiliations:** 1 Department of Onco-Hematology, Cell and Gene Therapies, Bambino Gesù Children’s Hospital, IRCCS, 00165 Rome, Italy; 2 Department of Imaging, Bambino Gesù Children’s Hospital, IRCCS, 00165 Rome, Italy; 3 Department of Pathology, Bambino Gesù Children’s Hospital, IRCCS, 00165 Rome, Italy; 4 Pediatric Radiotherapy Unit, Fondazione IRCCS Istituto Nazionale Tumori, 20133 Milan, Italy

**Keywords:** ganglioneuroblastoma, neuroblastoma, multifocal tumor

## Abstract

Neuroblastic tumors (NTs) represent the most common extracranial neoplasm occurring in childhood. Although ganglioneuroblastoma intermixed (GNBI) and ganglioneuroma (GN) are classified as very low-risk tumors, neuroblastoma (NB) and ganglioneuroblastoma-nodular (GNBN) may represent a serious risk to survival. Unfortunately, areas of GNBI and GNBN can coexist in the same mass, leading to incorrect risk staging when only biopsy is performed. Herein, we describe a case of multifocal NT (thoracic and abdominal localization) occurring in a 4-year-old male. Different histological subtypes, namely GNBI and GNBN, were revealed in the two lesions. We focus on the difficulties of proper diagnosis and risk stratification, underlining the usefulness of several diagnostic tools for appropriate management and therapeutic choices.

NTs are the most frequent extracerebral solid tumors in patients under 2 years of age and originate from sympathoadrenal lineage of neural crest-derived tissues; they develop most frequently in the abdomen, originating from the adrenal glands or paraganglia, in the thoracic, cervical and pelvic regions. According to the International Neuroblastoma Pathology Classification, four types of NTs are distinguished: NB, GNBN (composite, Schwannian stroma-dominant/stroma-rich and stroma-poor), GNBI (Schwannian stroma-rich) and GN (Schwannian stroma-dominant) maturing and mature subtypes [1,4].

Of note, GNBI usually has a good prognosis since it tends to differentiate and take on the histological and clinical features of GN, which represent the final stage of tumor maturation. Indeed, it has been hypothesized that “mature” NTs originally derive from a NB that has undergone differentiation and this is also suggested by the significantly higher mean age at diagnosis either in GN or in GNBI. [5,6,7]. GN and GNBI are always classified in the very low-risk group according to INRGSS, regardless of stage and age; treatment is surgical excision, which can be complete or partial [8,9]. Moreover, even in the presence of a neoplasm that is considered unresectable, chemotherapy is not recommended due to demonstrating a poor response with no decrease in tumor volume, and with increased toxicity [6,10]. Although GNBI is considered a benign pathology, it represents a gray area within the category of NTs; obtaining diagnostic certainty is, on the one hand, essential, but, on the other hand, difficult because it does not have radiological, metabolic and biochemical features of unambiguous interpretation. Conversely, GNBN can have a course similar to that of NB, requiring more aggressive therapeutic management. The INRGSS provides guidance for treatment choices based on the definition of different risk classes [11,12,13]; thus, it is crucial to obtain a definite diagnosis. For this reason, a surgical approach is often necessary, although burdened by possible postoperative complications. In fact, the biopsy sample may not be representative of the whole tumor, and the radiological and radiometabolic instrumental investigations do not yet provide diriment information to define a precise diagnosis [10]; for example, GNBN may be coexistent with ganglioneuromatous tissue and the nodular component, which is often necrotic or hemorrhagic, may not exhibit radiotracer uptake, leading to a negative result of ^123^I-MIBG scintigraphy. Finally, in large lesions with image-defined risk factors suspected to be GNBI, the dilemma is always whether to confirm the diagnosis with a biopsy that may be not non-representative of the entire tumor or to stress a debulking or major surgery that may present vital risk. Although histology is currently the only certain diagnostic mean in order to obtain the molecular information for prognostic definition, there is much debate about the role that radiological and nuclear medicine investigations might play in the diagnosis and differentiation of benign and malignant NTs [14]. Recently, few authors suggest the fundamental contribution of ^18^F-FDG PET being able to suggest NB or nodular variant and useful for subsequent clinical choices, in light of the poor contribution of ^123^I-MIBG scintigraphy in which ^123^I-MIBG uptake is present in mature as well as immature forms [15,16]. There is a correlation between ^18^F-FDG PET findings and disease status of NB which found that ^18^F-FDG PET uptake is directly proportional to tumor burden and tumor cell proliferation; therefore, higher values of SUVmax suggest the presence of a NT with unfavorable biological characteristics and worse prognosis [17]. Recently, meta-18F-fluorobenzylguanidine (^18^F-mFBG) showed promise for future staging and response assessment in NBL, allowing for fast and high-resolution imaging of tumors expressing the norepinephrine transporter [18]. Regarding MRI, reliability of DWI in the differential diagnosis of malignant and benign NT is much discussed and it was observed that lower ADC values are more characteristic of NB/GNBN than GNBI/GNs [14]. In the clinical case we describe, the absence of clinical and biochemical features that would point toward the diagnosis is peculiar; in fact, there is no increase in urinary catecholamines and the lesions show uptake at ^123^I-MIBG scintigraphy. In summary, the described case highlights the critical issues associated with the diagnosis of GNBN, which is a NT more similar to NB and a less mature form of GN/GNBI. Its diagnosis can be complex as there are no precise clinical and biochemical features that differentiate it from other NTs, and biopsy may not be representative of the whole tumor. Moreover, the presence of image-defined risk factors (IDRF) discourages aggressive surgery to confirm the histology variant and, considering the favorable GNBI/GNs outcome, the surgical risk may be considered and avoided. Significant progress is being made on the use of ^18^F-FDG PET and DWI, which could provide additional information on biological features, which can currently only be confirmed by histological examination but should be suggested by different imaging tools. According to our experience, the^18^F-FDG PET and DWI represent useful tools in “gray zone” GN/GNBI in order to avoid aggressive surgery with potential late sequels.

**Figure 1:**
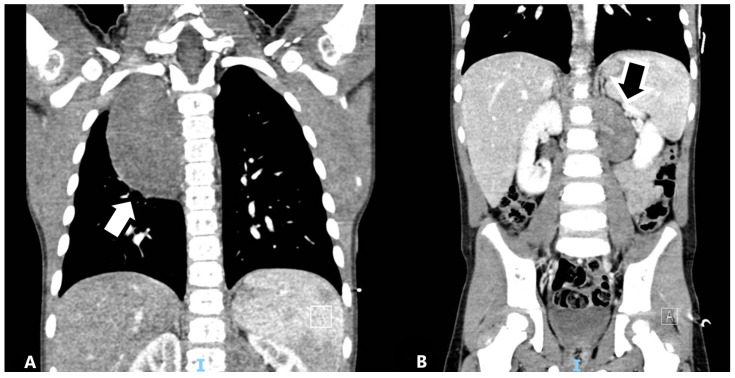
Computed tomography (CT) scan at diagnosis. The patient was a 4-year-old male with a delay in the achievement of normal developmental milestones as well as scoliosis. Difficulty walking was noted, so an X-ray of the spine was performed with the incidental finding of a mass located in the posterior mediastinum, making second-level imaging such as CT necessary. The images show the presence of a right paravertebral mediastinal mass (**A**) measuring 5.5 × 4.5 × 8 cm (corresponding to a volume of 99 mL), oval-shaped with homogeneous enhancement, and showed the presence of a second bilobed mass located at the left abdominal paravertebral level measuring 4.5 × 3.5 × 5.5 cm (corresponding to a volume of 43 mL) with similar CT features (**B**). At the thoracic level, the mass was in contact with the right main bronchus and the terminal part of the azygos vein, with no apparent infiltration of adjacent organs and structures; the abdominal lesion reached the kidney ileus with strict contact with both renal vein and artery.

**Figure 2:**
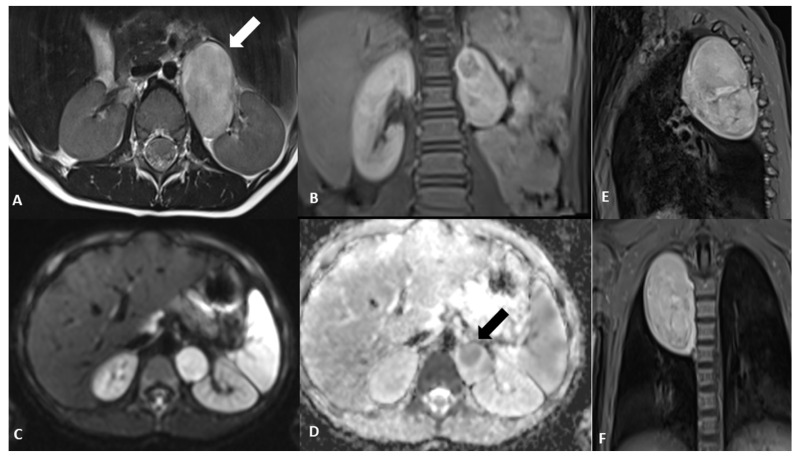
Magnetic resonance imaging (MRI) at baseline. The exam was performed in order to complete the framing and precisely define the anatomic relationships, which confirmed the two clear-edged, capsular, expansive formations located in the posterior mediastinum and in the left anterior paravertebral abdominal site, in the absence of intraspinal extension. An axial T2-weighted image shows the abdominal paravertebral mass (**A**) that presents inhomogeneous enhancement after contrast administration (**B**). The thoracic lesion in the posterior mediastinum shows a similar perfusion pattern, as shown in the coronal and sagittal T1-weighted images (**E**,**F**). (**C**) MRI axial diffusion-weighted imaging (DWI) shows a hyperintense rounded area with low apparent diffusion coefficient (ADC) value in the upper portion of the abdominal mass, as shown in (**D**) (arrow). Blood tests and urinary catecholamines were within normal limits. In order to determine a diagnosis of certainty, an echo-guided biopsy of the lesion located at the level of the posterior mediastinum was performed, as it was more easily reached than the abdominal mass; histopathological features were finally consistent with a GNBI, with the presence of Schwannian stromal development occupying >50% of tumor tissue [1] (see also histological image).

**Figure 3:**
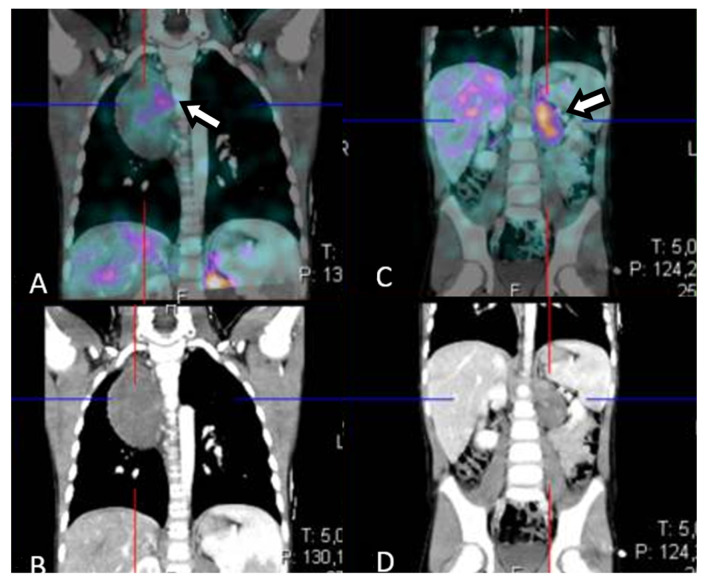
Scintigraphy with ^123^I- meta-iodobenzylguanidine (^123^I-MIBG) scan at baseline. To stage the tumor, a ^123^I-MIBG scan was performed, showing diffuse slight uptake in the thoracic mass (**A**,**B**) and an inhomogeneous uptake pattern in the abdominal mass (**C**,**D**) without metastatic lesions. A bone marrow aspiration was performed to complete staging and showed no evidence of neuroblastic cells. Based on the radiological features and according to the INRG Staging System (INRGSS), the mediastinal and abdominal lesions were defined as an L2 stage [2]. In view of the GNBI diagnosis (a low-risk tumor), the critical location of the neoplasms and the total absence of clinically evident symptoms, a watch-and-wait strategy was chosen [1,3].

**Figure 4:**
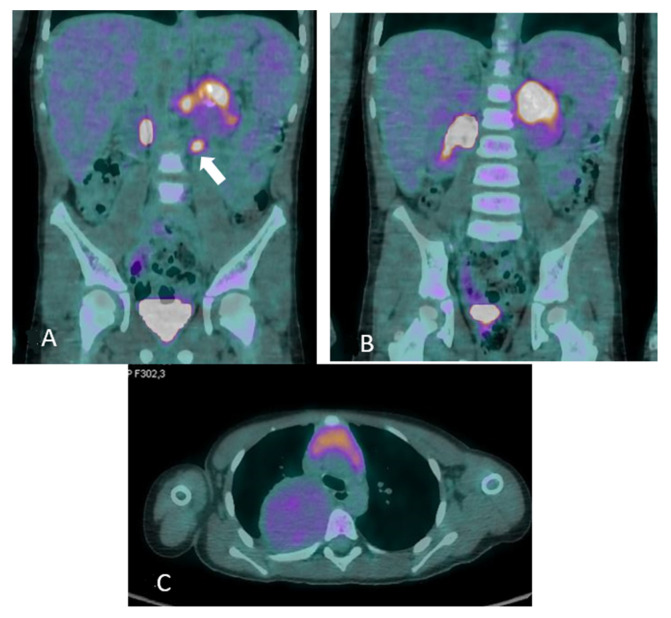
^18^F-fluorodeoxyglucose/computed tomography (^18^F-FDG PET/CT) at one year from diagnosis. The patient was in good general clinical condition and the disease was stable; however, there were critical issues that were determined by the inability to rule out a nodular component within the already biopsied thoracic mass or the unstudied abdominal mass (see Figure 7). In order to characterize the metabolic activity of the mass, a ^18^F-FDG PET/CT scan was performed and showed FDG uptake in the left paravertebral mass (**A**) (maximum standardized uptake value -SUVmax-6.0), in the para-aortic lymph nodes (**B**) (maximum size 1.2 cm, SUVmax 5.8) and in the thoracic mass (**C**) (SUVmax2.3); no tracer uptake was detected in other sites. Tumor markers remained stable and within normal limits at all evaluations. The radiological and metabolic features assessed by ^18^F-FDG PET have not allowed the nature of the lesions to be clearly defined; in particular, with the available radiological investigations, the differentiation of benign and malignant NTs is quite controversial so far. Nevertheless, the different FDG uptake of abdominal lesions with lymph node involvements suggests a nodular component. In addition, the biopsy performed at onset may have been unrepresentative of the whole tumor, leading to late misdiagnosis with significant clinical fallout.

**Figure 5:**
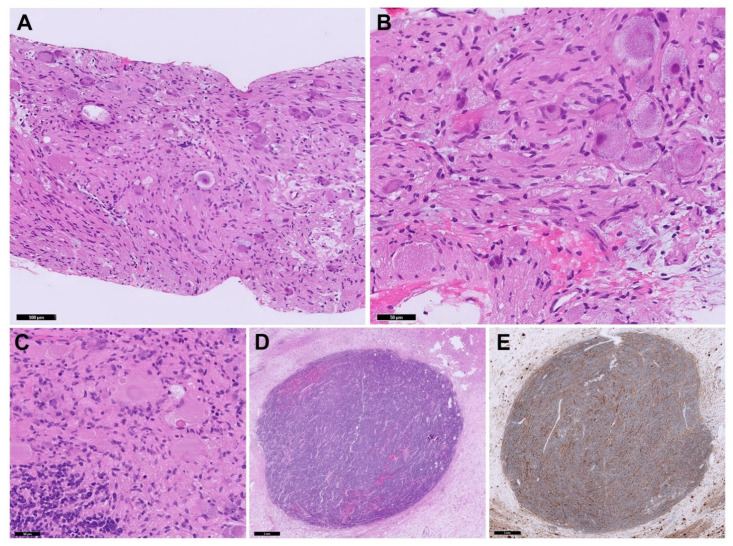
Microscopic examination of the neoplasm. On the increased uptake found at ^18^F-FDG PET in the abdominal mass, the child underwent laparotomic partial excision of the abdominal mass: (**A**,**B**) Needle core biopsy of the thoracic lesion showed a GNBI morphology (H&E stain); (**C**) Surgical excision of the abdominal mass revealed a GNBN morphology showing well-defined nodules (**D**) of Schwannian stroma-poor NB, poorly differentiated (H&E stain). (**E**) Synaptophysin immunoperoxidase stain highlighting stroma-poor nodules. In addition, NB undifferentiated foci were documented in a lymph node. Restaging included a bone marrow aspiration and biopsy, which ruled out osteo-medullary infiltration.

**Figure 6:**
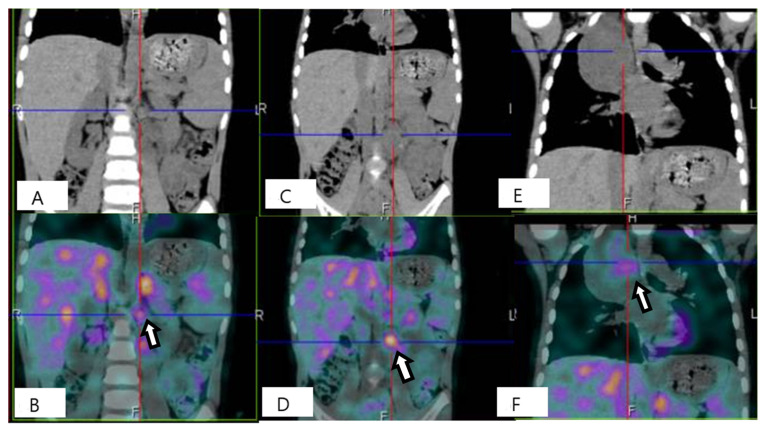
Reevaluation with ^123^I-MIBG scintigraphy confirmed the presence of uptake at both abdominal (**A**–**D**) and thoracic level without metastatic lesions (**E**–**F**).

**Figure 7:**
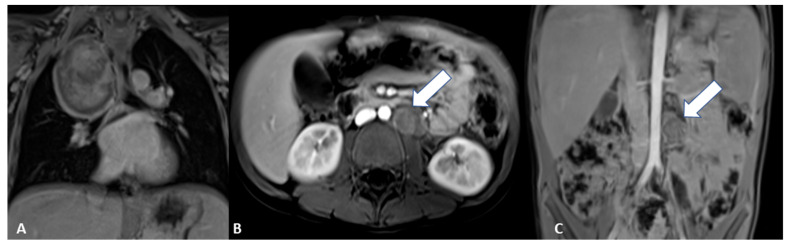
Reevaluation with MRI showed the thoracic lesion with unchanged features (**A**), and a residual tumor >2 cm at abdominal lesion (**B**) with a local enlarged lymph node (**C**). Clinical (L2 stage and age >18 months), histological and molecular features (MYCN not amplified and presence of SCAs) led to classification into the intermediate risk group according to the Low/Intermediate risk Neuroblastoma European Study (LINES) [2].

## Data Availability

The data presented in this study are available on request from the corresponding author.
